# Supplementary figures and images for: Inhibition of IL-34 Unveils Tissue-Selectivity and Is Sufficient to Reduce Microglial Proliferation in a Model of Chronic Neurodegeneration
Source: Front Immunol. 2020 Oct 8;11:579000. doi: 10.3389/fimmu.2020.579000 (PMC7580706; doi:10.3389/fimmu.2020.579000)

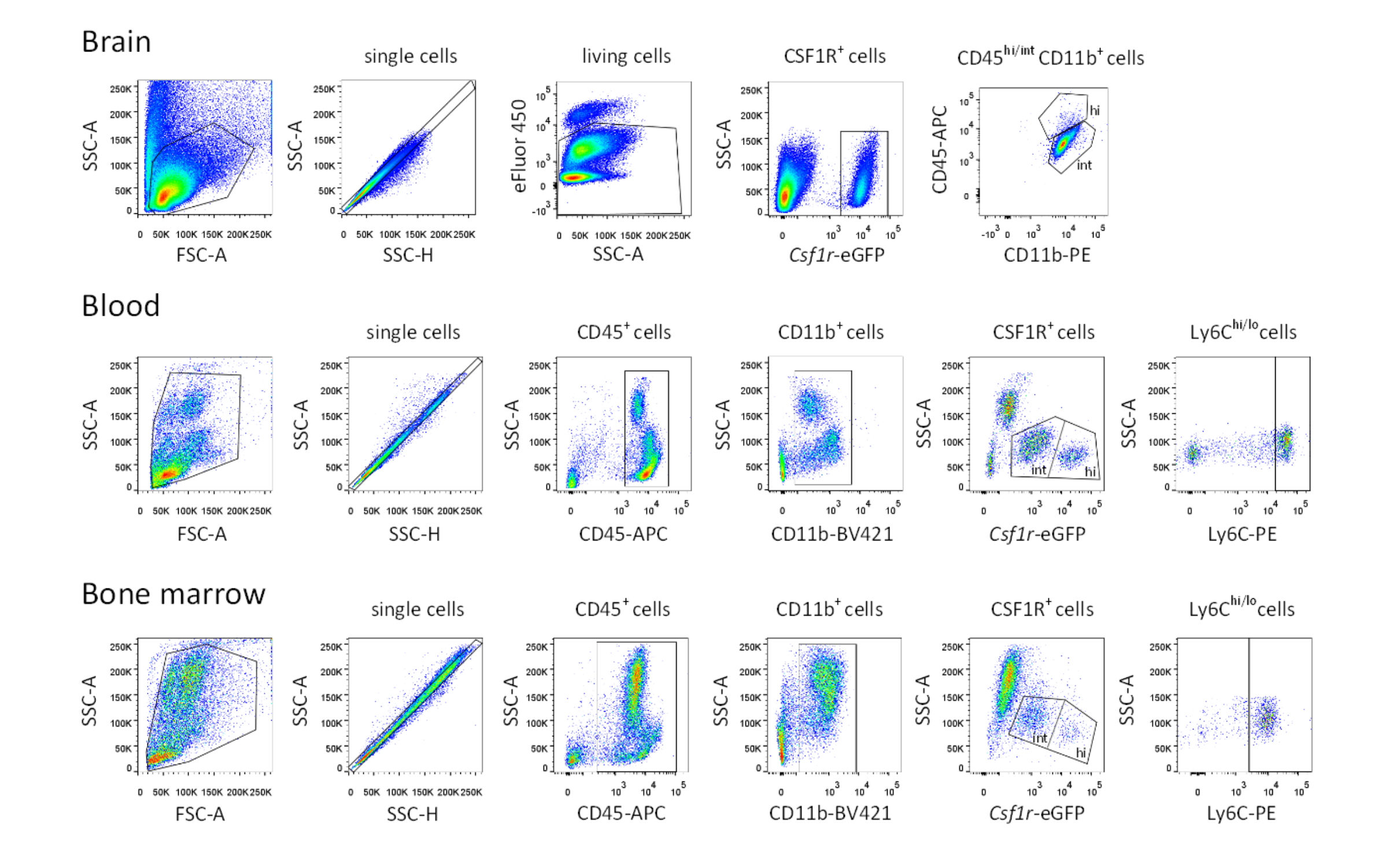

Supplement: Supplementary Figure 1 — Gating strategy used for flow cytometric analysis of brain, blood, and bone marrow after CSF1R- and IL-34 antibody treatment. [file Image_1.TIF]

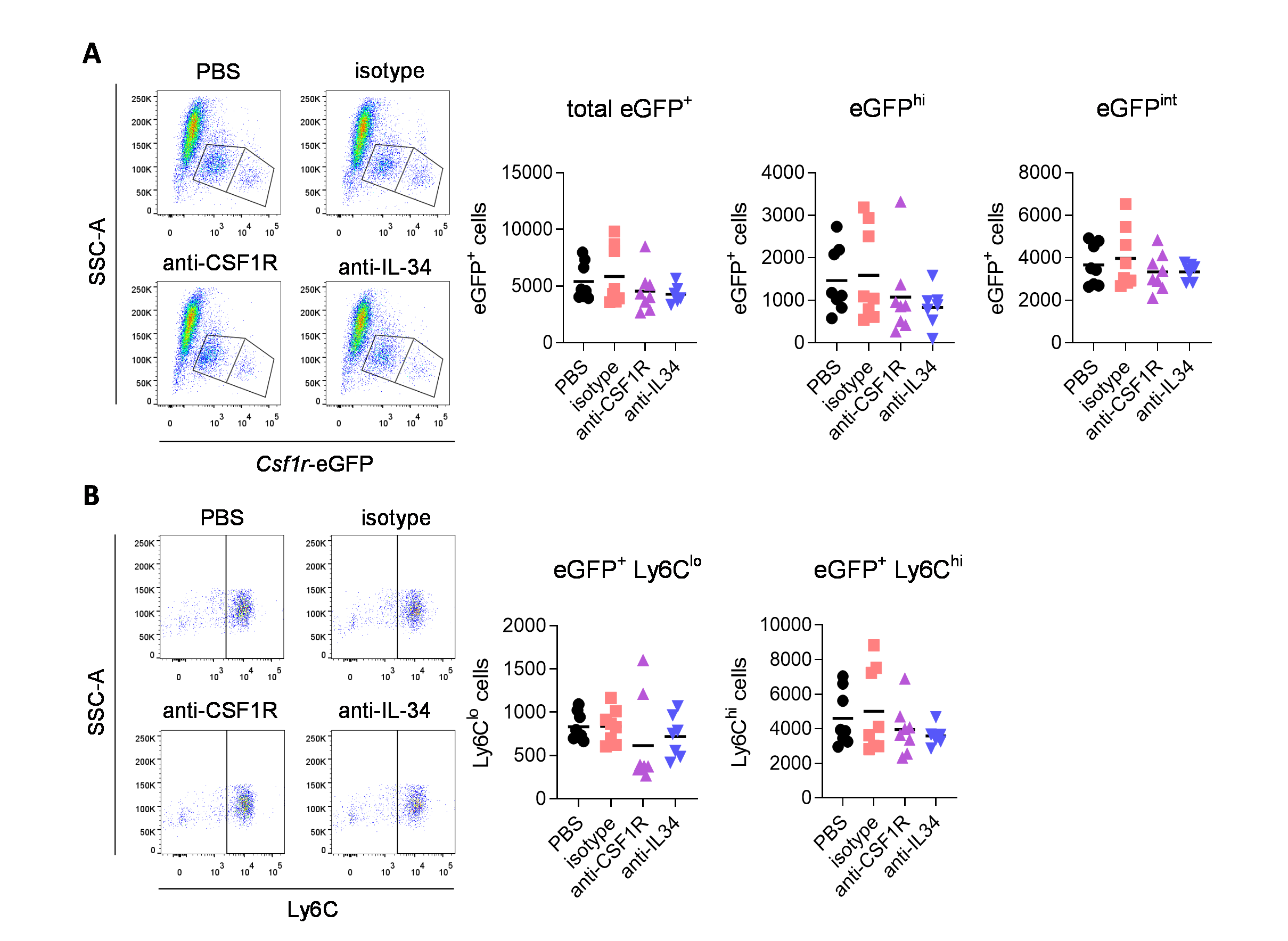

Supplement: Supplementary Figure 2 — Effect of CSF1R- and IL-34 antibody treatment on bone marrow. Macgreen mice were treated with anti-CSF1R or anti-IL-34 (both rat monoclonal IgG2A) by intraperitoneal injections of 250 μg antibody 3x a week for 3 weeks. (A) Flow cytometric analysis of CSF1R+ cells in the bone marrow of anti-CSF1R- and anti-IL-34- treated mice did not result in significant alterations. Graphs indicate respective cell numbers per 5 × 10^4^ CD45+ cells. (B) Flow cytometry of eGFP+ Ly6Chi and eGFP+ Ly6Clo subpopulations of CSF1R-expressing cells in the bone marrow did not reveal any significant changes due to the treatment. Graphs indicate respective cell numbers per 5 × 10^4^ CD45+ cells. PBS n = 8, isotype n = 8, anti-CSF1R n = 8, anti-IL-34 n = 7, data shown represent mean ± SEM, two-way ANOVA followed by Tukey's multiple comparison test. *p < 0.05, **p < 0.01, ***p < 0.001. [file Image_2.tif]

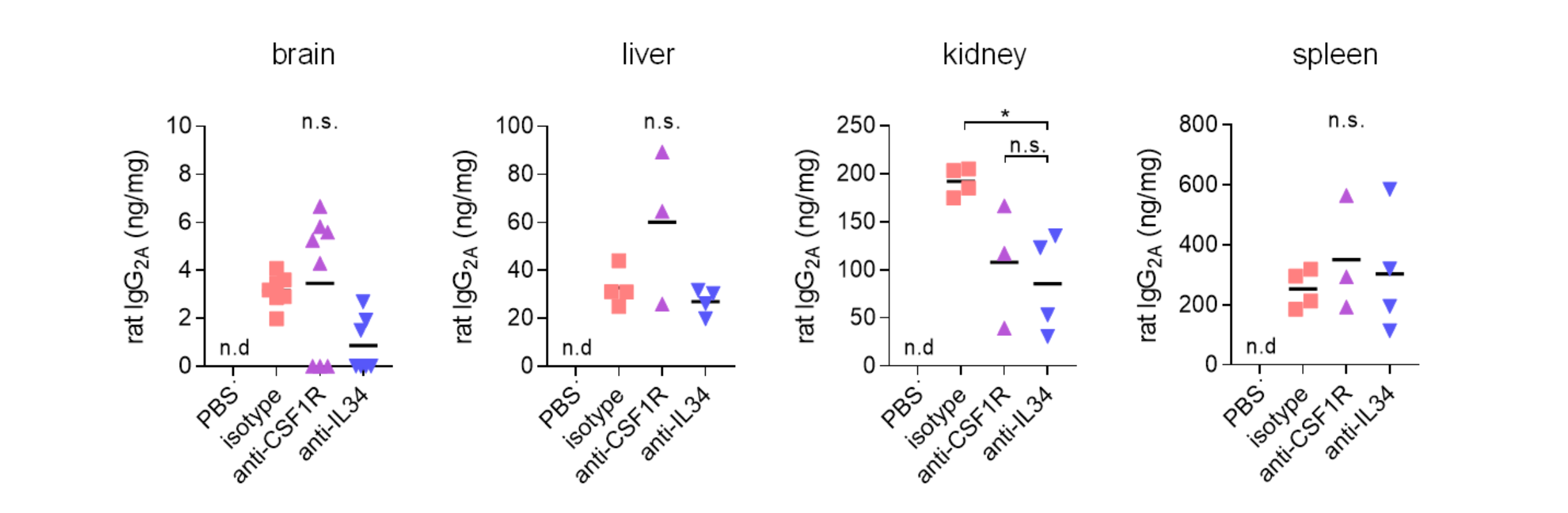

Supplement: Supplementary Figure 3 — Distribution of CSF1R- and IL-34 antibodies in peripheral organs and brain. Macgreen mice were treated with anti-CSF1R or anti-IL-34 (both rat monoclonal IgG2A) by intraperitoneal injections of 250 μg antibody 3x a week for 3 weeks. Levels of rat IgG2a were measured in tissue lysates of brain, liver, kidney and spleen after the treatment by ELISA, showing no significant differences between anti-CSF1R and anti-IL-34 in individual organs. Brain: PBS n = 8, isotype n = 8, anti-CSF1R n = 8, anti-IL-34 n = 7, liver/kidney/spleen: PBS n = 4, isotype n = 4, anti-CSF1R n = 3, anti-IL-34 n = 4, data shown represent mean ± SEM, two-way ANOVA followed by Tukey's multiple comparison test. *p < 0.05, **p < 0.01, ***p < 0.001. [file Image_3.tif]
